# Medical Students’ Corner: Barriers to Communication During the COVID-19 Pandemic

**DOI:** 10.2196/24989

**Published:** 2020-11-27

**Authors:** M Olabisi Ogunbiyi, Emma Obiri-Darko

**Affiliations:** 1 University College London Medical School London United Kingdom

**Keywords:** COVID-19, medical education, education, student, communication, perspective, medical student, barrier, culture

## Abstract

The COVID-19 pandemic has inspired us, as medical students, to reflect upon the communication training we have received in medical school and the obstacles we have faced in the clinic due to COVID-19. We hold the view that our communication training is inadequate; this view is driven by our limited exposure to patients, a situation that is currently being exacerbated by the pandemic. The medical curriculum must be inclusive of all groups and take into account the new challenges arising during the COVID-19 pandemic.

Communication is vital in improving health outcomes, especially in marginalized communities such as non-English speakers and people with impaired hearing. Indeed, patients from these groups have lower satisfaction and outcomes in most health care settings [1,2]. Currently, medical education is lacking in providing students with the necessary skills to facilitate adequate care for people in these communities. These skills include verbal and nonverbal communication, cultural sensitivity, adapting the clinical environment, and accessing medical translation facilities. Our medical training does not include any consultation models geared specifically towards patients who are deaf or who are not fluent in English. We also do not receive simulation training on how to communicate with these patients or on how to access translation or sign language services during placements. In our own experience, we often feel unable to take a complete history, and we often wonder if the patient fully understands our advice.

The use of masks is now widespread in every health care setting. This can be an unsettling communication barrier, especially for people with hearing impairment or limited English language skills [3,4]. The use of face masks also has a detrimental effect on information exchange, shared decision-making, and patient adherence to medical advice [5]. Although masks help reduce disease transmission, little consideration is given to patients who rely on nonverbal communication to navigate their health care. We have found that with masks, it is difficult to gauge the patient’s emotions, and it is even more difficult for the patient to understand us. We propose the introduction of simulation training with people with impaired hearing to develop the skills necessary to provide an adequate consultation. In addition, transparent surgical masks should be made available in every health care setting for this purpose [3]. Medical sign language could also be offered as an extracurricular component in medical schools.

To minimize infection exposure, the use of telephone consultations in primary care has increased dramatically [5]. General practitioners are advised to reserve face-to-face appointments for acutely ill patients only. This approach poses several barriers to people with language difficulties. First, in our experience, many patients opt out of or refuse video consultations, which minimizes the use of visual aids, gestures, and drawings—techniques we are encouraged to use in our medical school training. Indeed, as medical students, we feel ill-equipped to conduct telephone consultations with people who are not fluent in English, as we cannot use visual cues or body language to communicate. We have found that we are often misunderstood by patients during telephone consultations; also, patients are often too embarrassed to tell us they do not understand. These challenges will continue to be present during the pandemic as the number of telephone consultations continues to increase and as such consultations become a more permanent fixture in many health care settings. This exemplifies the need for simulation training on both remote consulting and consulting with people with limited proficiency in English. Additionally, video consultations should be enforced where necessary for adequate communication.

Due to the new challenges posed by the COVID-19 pandemic, already ill-equipped students are encountering more challenges. It should also be noted that many medical students were away from the clinical setting for almost 6 months, further reducing their ability to practice and develop communication skills. Our current curriculum only includes a few simulation sessions in the entire course; these sessions feature monolingual English-speaking actors with unimpaired hearing. Based on student feedback, we encourage medical schools to provide more virtual sessions to teach communication and also to introduce teaching tailored to consider mask-wearing and language barriers. We hope this will become a core facet of the medical school curriculum. We believe that establishing this form of teaching has the potential to shape a generation of medical students who are skilled at communicating with a diverse range of people. If this gap in health care is not urgently addressed, it will only widen.

## References

[1] Egede LE (2006). Race, ethnicity, culture, and disparities in health care. J Gen Intern Med.

[2] Kuenburg A, Fellinger P, Fellinger J (2016). Health Care Access Among Deaf People. J Deaf Stud Deaf Educ.

[3] Atcherson SR, Mendel LL, Baltimore WJ, Patro C, Lee S, Pousson M, Spann MJ (2017). The Effect of Conventional and Transparent Surgical Masks on Speech Understanding in Individuals with and without Hearing Loss. J Am Acad Audiol.

[4] Chodosh J, Weinstein BE, Blustein J (2020). Face masks can be devastating for people with hearing loss. BMJ.

[5] (2020). Appointments in General Practice August 2020. NHS Digital.

